# Supplementary figures and images for: Recurrent connections facilitate symmetry perception in deep networks
Source: Sci Rep. 2022 Dec 3;12:20931. doi: 10.1038/s41598-022-25219-w (PMC9719566; doi:10.1038/s41598-022-25219-w)

InceptionResNetV2

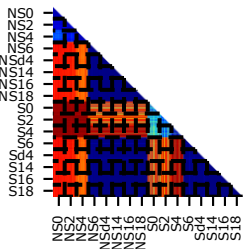

InceptionV3

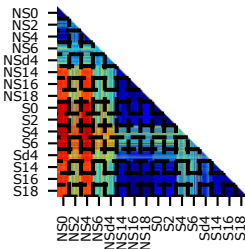

ResNet101

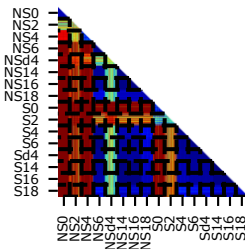

DenseNet

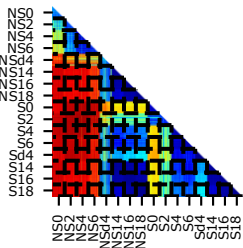

Dilated

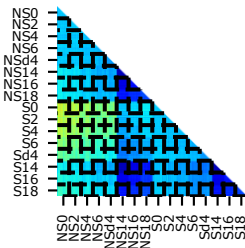

Xception

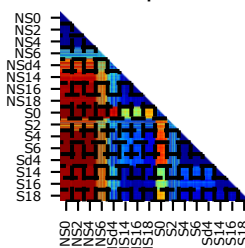

Transformer

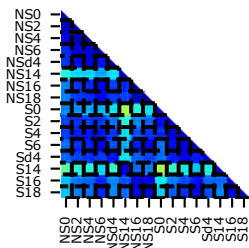

LSTM3

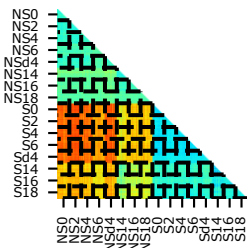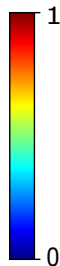

Supplement: Supplementary file 1 — Supplementary Figure 1. [file 41598_2022_25219_MOESM1_ESM.pdf]
